# Population‐Based Cohort Study for Development of National Retinopathy of Prematurity Screening Criteria

**DOI:** 10.1111/apa.70381

**Published:** 2025-11-25

**Authors:** R. Gerull, C. Sanchez, A. Atkinson, M. Schuler‐Barazzoni, M. Adams, F. Barcos Munoz, C. Gerth‐Kahlert, S. M. Schulzke, the Swiss Neonatal Network, Ph. Meyer, L. Eisenhut, M. Bryant, A. Kidszun, T. Riedel, M. Gebauer, B. Rogdo, B. Wagner, R. E. Pfister, J.‐F. Tolsa, J. Schneider, M. Stocker, P. Gessler, B. Laubscher, J. Llor, A. Malzacher, A. Birkenmaier, L. Hegi, V. Bernet, M. Tomaske, D. Bassler, V. Cannizzaro, C. Hagmann

**Affiliations:** ^1^ Department of Neonatology University Children's Hospital Basel UKBB Basel Switzerland; ^2^ Faculty of Medicine University of Basel Basel Switzerland; ^3^ Pediatric Research Centre, University Children's Hospital Basel, University of Basel Basel Switzerland; ^4^ Division of Infectious Diseases & Institute for Informatics, Data Science, and Biostatistics, Washington University in St. Louis School of Medicine St. Louis Missouri USA; ^5^ Clinic of Neonatology, Department Woman‐Mother‐Child, University Hospital of Lausanne Lausanne Switzerland; ^6^ Department of Neonatology, Newborn Research University Hospital Zurich (USZ), University of Zurich (UZH) Zurich Switzerland; ^7^ Neonatal Intensive Care Unit University Hospital of Geneva Geneva Switzerland; ^8^ Department of Ophthalmology, University Hospital Zurich and University of Zurich Zurich Switzerland

**Keywords:** preterm, retinopathy of prematurity, screening

## Abstract

**Aim:**

Screening criteria for retinopathy of prematurity (ROP) vary among countries. Early detection of ROP and minimising the burden of screening are important.

**Methods:**

We analysed data from very preterm infants born in Switzerland between 2006 and 2022. Logistic regression models were fitted to evaluate 17 potential risk factors for ROP treatment.

**Results:**

168/11354 patients (median (range) gestational age (GA) 29.6 (23.0–31.9) weeks) required ROP treatment. All would have been detected and screening burden would have been reduced by 56% if screening had required meeting ≥ 1 of the following criteria: GA < 27 weeks (89.3%), birth weight < 1000 g (97.0%), intraventricular haemorrhage≥II° (24.0%), congenital tumour (1.2%). We identified six statistically significant risk factors for ROP: GA (adjusted odds ratio (aOR) 0.46, 95% CI 0.40–0.52, *p* < 0.001), birth weight z‐score (aOR 0.58, 95% CI 0.46–0.73, *p* < 0.001), duration of supplemental oxygen (aOR 1.01 95% CI 1.01–1.02, *p* < 0.001), duration of mechanical ventilation (OR 1.01, 95% CI 1.00–1.02, *p* = 0.018), caesarean section (OR 1.84, 95% CI 1.06–3.36, *p* = 0.038), and congenital tumour (OR 26.3, 95% CI 2.71–189, *p* = 0.002). The model allowed for excellent prediction of ROP treatment (AUC 0.963, 95% CI 0.944–0.981).

**Conclusions:**

Safely reducing the burden of ROP screening appears achievable in Switzerland.

**Abbreviations**
aOR: adjusted odds ratio
AUC: area under the curve
CPAP: continuous positive airway pressure
GA: gestational age
IQR: interquartile Range
IVH: intraventricular haemorrhage
NEC: necrotizing enterocolitis
PDA: patent ductus arteriosus
PMA: postmenstrual age
ROC: receiver operating characteristic curve
ROP: Retinopathy of Prematurity
SGA: small for gestational age
SNN: Swiss Neo Net
VEGF: vascular endothelial growth factor

**Summary**

- The incidence of retinopathy of prematurity (ROP) treatment in Switzerland is lower than in other high‐income countries, warranting national ROP screening criteria.
- Six risk factors allowed for excellent statistical prediction of ROP treatment in multivariable logistic regression analysis.
- The combination of four clinical parameters (GA < 27 0/7 weeks, birthweight < 1000 g, intraventricular haemorrhage ≥II, congenital tumour) identified all patients requiring ROP therapy and allows to reduce the burden of screening by 56%.

## 1 Introduction

Retinopathy of prematurity (ROP) is a vascular proliferative disorder of the retina, which exclusively develops in preterm infants due to immaturity of the retinal vascularization [1]. The underlying pathophysiological mechanisms are mainly driven by a mismatch between oxygen demand and supply, leading to pathologic vascularization of the retina which may ultimately result in retinal detachment or even blindness if untreated [2, 3]. In fact, ROP is a leading cause of childhood blindness worldwide [4]. The incidence of ROP varies, with a higher incidence in low‐and middle‐income countries, where more mature infants are affected compared to high‐income countries [5]. However, significant differences in ROP incidence have also been shown between high‐income countries [6].

ROP screening is essential for detecting ROP development. It enables timely treatment of pathologic retinal vascularization, helping to prevent severe visual impairment or blindness. However, ophthalmoscopic examination of the eye is associated with pain and stress for preterm infants [7, 8]. There is no direct evidence that ophthalmoscopy leads to neurodevelopmental impairment. On the other hand, repetitive stress and pain may be associated with adverse long‐term neurodevelopment [9]. Thus, the benefits of early detection of ROP should be balanced with the burden of repeated retinal examination in this vulnerable population.

Worldwide variations in ROP incidence require that ROP screening strategies are adapted to local conditions to avoid unnecessary screening on the one hand and possible blindness on the other. This is reflected in the wide variations of screening criteria across national screening programs, with more liberal criteria in low‐and middle‐income countries [5, 10].

Currently, no national guideline for ROP screening exists in Switzerland. Given that Switzerland has a lower ROP incidence than other high‐income countries [6], adopting screening criteria from other countries may lead to unnecessary screening. In turn, this may result in excess pain and stress for preterm infants along with increased clinical workload and financial costs.

The purpose of this study was to analyse registry data from very preterm infants born in Switzerland with the aim of developing a national guideline for ROP screening.

## 2 Material and Methods

We conducted a retrospective cohort analysis of preterm infants born between 2006 and 2022 in Switzerland and who were registered in the national registry of very preterm infants (Swiss Neonatal Network, SwissNeoNet). This network prospectively collects a routine set of perinatal and follow‐up data of live born infants with a gestational age (GA) between 22 0/7 and 31 6/7 weeks or a birth weight ≤ 1500 g [11]. Additionally, the network collects routine follow‐up data at 18 to 24 months postmenstrual age (PMA). All nine Swiss perinatal centers and most step‐down units participated. Parents or guardians were informed about the use of data for research purposes. Moreover, this study was approved by the cantonal ethics committee of Basel (EKNZ2023‐01288). Routine comparison with the Swiss Federal Statistical Office revealed population coverage comparable with the Swiss vital statistics.

All patients registered in the SwissNeoNet (SNN) database who were born 2006–2022 were eligible for this study. Exclusion criteria were GA ≥ 32 0/7 weeks, primary palliative care (including all patients < 23 0/7 weeks GA according to Swiss recommendations), patients who died during primary hospitalisation and those with missing data in any of the relevant risk factors.

Baseline patient characteristics were summarized as frequency (%) for categorical variables and median (interquartile range [IQR]) for continuous variables unless stated otherwise.

The following 17 potential risk factors for ROP treatment were available from the SNN database and included in our analyses: caesarean section, antenatal corticosteroids, sex, GA, small for gestational age (SGA) (< 10th percentile according to Fenton [12]), multiple birth, birth weight *z*‐score, delivery room endotracheal intubation, surfactant treatment, days of supplemental oxygen (saturation target range according to local protocol), days of continuous positive airway pressure (CPAP), days of mechanical ventilation, intraventricular haemorrhage (IVH) ≥ II° classified according to Papile et al. [13], congenital tumour (excluding simple hemangioma), patent ductus arteriosus (PDA), sepsis (culture positive or clinical culture negative sepsis), and necrotizing enterocolitis (NEC) Bell stages ≥ II [14]. Data on the timing of ROP treatment were collected based on PMA and chronological age (days of life) at ROP therapy.

Logistic regression with and without multiple imputation was used to identify the best‐performing model. Patients with missing data in any of the relevant risk factors were excluded. The dataset was divided into training and test in an 80:20 proportion. To ensure robust evaluation of model performance and mitigate overfitting, we employed fivefold cross‐validation during the training process. The dataset was randomly partitioned into 5 equal‐sized subsets (folds). For each iteration, one fold was held out as the validation set, while the remaining four folds were used for training the model. This process was repeated five times, ensuring that each fold served as the validation set exactly once. Predictive models were evaluated on the test dataset using the area under the receiver operating characteristic curve technique. Forward‐backward variable selection was used to identify the most parsimonious model. Variables with a *p* < 0.2 in univariable regression were considered for the stepwise elimination process. The final model included GA, birthweight z‐score, duration of supplemental oxygen, duration of mechanical ventilation, caesarean section, and congenital tumour as risk factors. Analyses were performed with the statistical software R (version 4.2.1, R Development Core Team, Vienna, Austria) [15].

Identified ROP screening criteria were compared to current German and American guidelines, which state that general ROP screening is recommended for infants < 31 0/7 weeks GA or birthweight < 1500 g [16, 17].

## 3 Results

**FIGURE 1:**
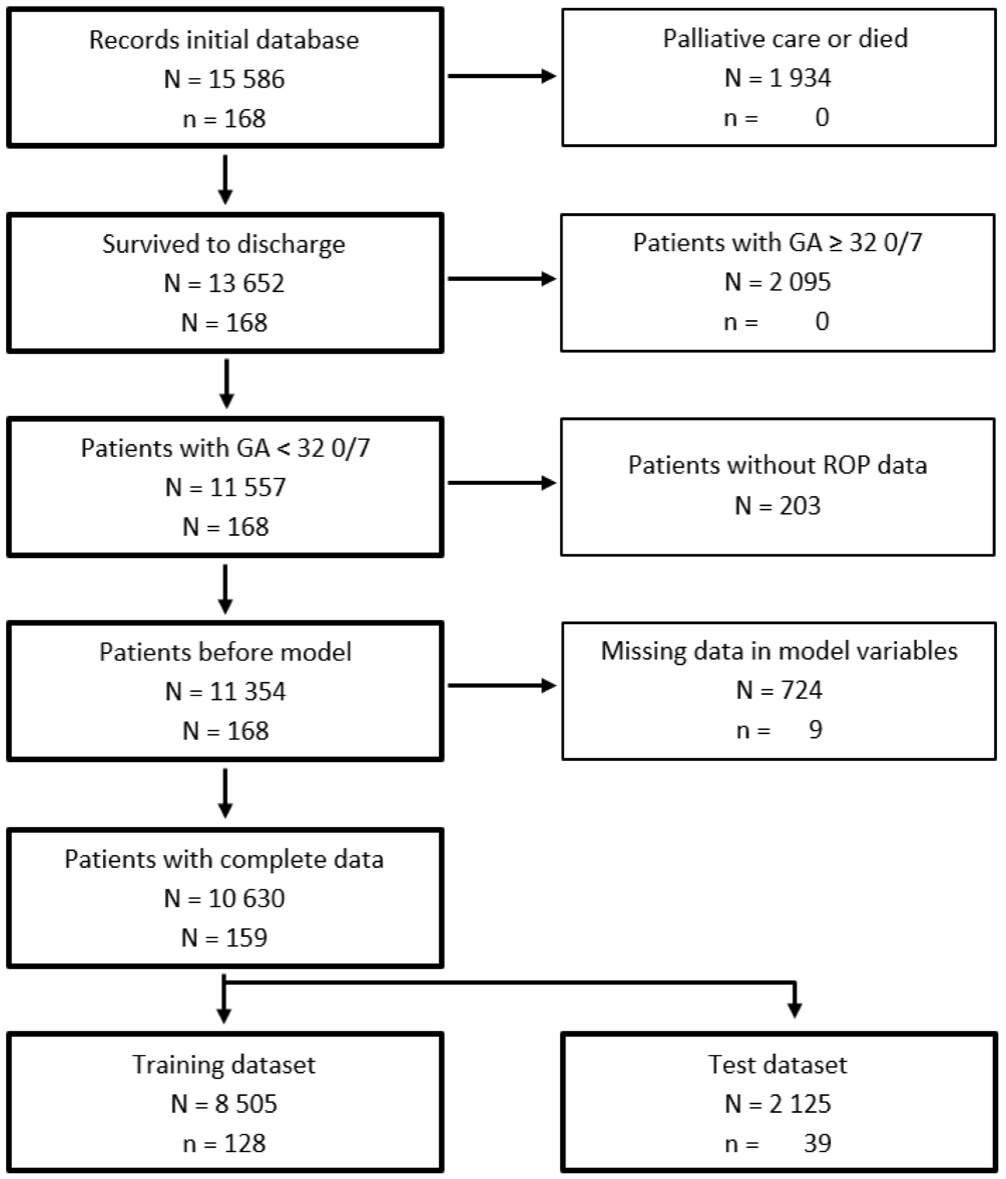
Flow chart of patient selection and exclusion along with reasons. *N* = total number of patients, *n* = number of patients who required ROP treatment.

Between 2006 and 2022, 15 586 patients were registered in SNN, covering 98% of Swiss patients in the target population, making the analyses representative for Switzerland. Of these, 4232 did not meet the inclusion criteria (Figure 1) and 11 354 patients remained for further analyses.

Patients included in this study had a median (interquartile range; range) GA of 29.6 (27.7, 31.0; 23.0, 31.9) weeks and a birth weight of 1200 (935, 1490; 300, 2670) g. Detailed patient characteristics are shown in Table 1. While patients with congenital tumours and ROP treatment showed nephroblastoma (*n* = 1; 30 1/7 weeks GA, birth weight 1530 g) and congenital capillary malformation syndrome (*n* = 1; 29 0/7 weeks GA, birth weight 1250 g), patients with tumour and no ROP therapy had sacrococcygeal teratoma (*n* = 3), large and multiple hepatic hemangioma (*n* = 1), tracheal hemangioma (*n* = 1), Sturge–Weber or Klippel‐Trenaunay Syndrome with large cutaneous hemangioma (*n* = 1), melanocytic tumour (*n* = 1), cardiac tumour (*n* = 1) and spinal bone tumour (*n* = 1).

**TABLE 1:** Patient characteristics.

	Overall, *n* = 11 354	No ROP therapy, *n* = 11 186	ROP therapy, *n* = 168	*p* ^a^
Gestational age (weeks)^b^	29.6 [27.7, 31.0]	29.7 [27.9, 31.0]	25.3 [24.4, 26.0]	< 0.001
Antenatal steroids	10 320 (92.3%)	10 164 (92.3%)	156 (92.9%)	0.8
Multiple birth	3895 (34.3%)	3844 (34.4%)	51 (30.4%)	0.3
C‐section	9061 (80.2%)	8914 (80.1%)	147 (88.0%)	0.010
Sex (male)	6084 (53.6%)	5988 (53.5%)	96 (57.1%)	0.4
Birth weight^b^ (g)	1200 [935, 1490]	1215 [950, 1490]	650 [550, 746]	< 0.001
Birthweight *z*‐score^b^	−0.1 [−0.6, 0.4]	0.0 [−0.6, 0.4]	−0.6 [−1.4, 0.0]	< 0.001
SGA	952 (8.4%)	905 (8.1%)	47 (28.0%)	< 0.001
Intubation	3536 (32.5%)	3420 (31.9%)	116 (69.9%)	< 0.001
Surfactant	5320 (47.0%)	5177 (46.5%)	143 (85.1%)	< 0.001
Supplemental oxygen (days)^b^	4.0 [1.0, 31.8]	4.0 [1.0, 30.0]	78.5 [58.8, 102.0]	< 0.001
CPAP (days)^b^	10.0 [3.0, 33.0]	9.0 [3.0, 32.0]	46.0 [34.5, 59.0]	< 0.001
Mechanical ventilation (days)^b^	0.0 [0.0, 2.0]	0.0 [0.0, 2.0]	21.0 [7.5, 33.0]	< 0.001
Sepsis	3346 (29.6%)	3235 (29.0%)	111 (66.5%)	< 0.001
NEC	487 (4.3%)	457 (4.1%)	30 (17.9%)	< 0.001
IVH	2045 (18.1%)	1983 (17.8%)	62 (37.1%)	< 0.001
PDA	2720 (24.0%)	2602 (23.4%)	118 (70.2%)	< 0.001
Congenital tumour	11 (0.1%)	9 (0.1%)	2 (1.2%)	0.011

Abbreviations: IVH, intraventricular haemorrhage; NEC, necrotizing enterocolitis; PDA, significant patent ductus arteriosus; SGA, small for gestational age (< 10th percentile).

^a^ Wilcoxon rank sum test; Pearson's chi‐squared test; Fisher's exact test.

^b^ Median [IQR]; *n* (%).

Overall, 168 patients (1.5%) required ROP treatment. As expected, ROP treatment decreased with increasing GA (Table 2). Patients born at 23 completed weeks GA had a risk of 16.1% for ROP treatment, while the risk was minimal for patients above 27 weeks GA.

**TABLE 2:** ROP treatment by: (a) gestational age at birth (completed weeks) and (b) birth weight (g). Right value in interval included.

GA (weeks)	Patients (*n*)	ROP treatment (*n*)	ROP treatment (%)
(a)			
23	62	10	16.1
24	374	60	16.0
25	600	50	8.3
26	913	30	3.3
27	1129	7	0.6
28	1411	7	0.5
29	1739	2	0.1
30	2247	2	0.1
31	2879	0	0.0
All	11 354	168	1.5

Birth weight (g)	Patients (*n*)	ROP treatment (*n*)	ROP treatment (%)
(b)			
≤ 500	135	31	23.0
(500–750]	1253	96	0.9
(750–1000]	2312	36	1.6
(1000–1250]	2482	4	0.2
(1250–1500]	2550	0	0
> 1500	2621	1	0
All	11 354	168	1.5

Descriptive analyses revealed that all patients requiring ROP treatment would have been identified if patients meeting at least one of the following four parameters had been screened: GA < 27 0/7 weeks, birth weight < 1000 g, IVH≥II°, and congenital tumour. Applying these parameters to select patients for screening would have reduced the number of patients screened by 56% compared to current German and American guidelines.

**FIGURE 2:**
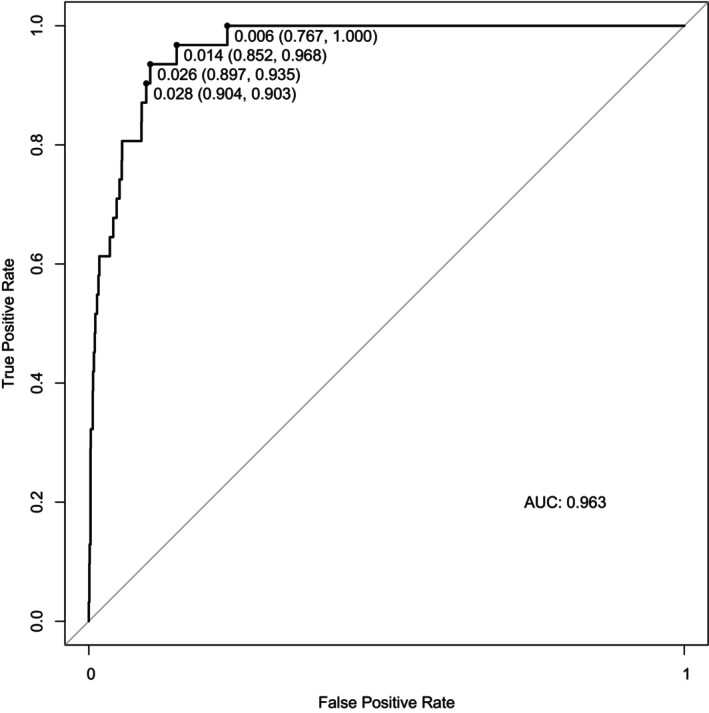
Receiver operating characteristics (ROC) curve of the final model. Included risk factors: GA, birth weight *z*‐score, days of supplemental oxygen, days of mechanical ventilation, congenital tumour, c‐section.

Six percent of the patients were removed (724/11 354) due to missing data during the creation of the model. There were no statistically significant differences between included and excluded patients. The training dataset included 8505 patients, while the test dataset included 2125 patients. Logistic regression analysis identified 6 parameters as independent risk factors for ROP treatment: GA, birthweight z‐score, duration of supplemental oxygen, duration of mechanical ventilation, caesarean section, and congenital tumour (Table 3). The other 11 risk factors were discarded during stepwise elimination in the multivariable forward–backwards variable selection process. The combination of identified risk factors allowed excellent prediction of ROP treatment with an area under the curve (AUC) of 0.963 (95% CI 0.944–0.982) in receiver operating characteristics curve (ROC) (Figure 2). An optimal cutoff for the logistic regression of 0.006 was identified from the model which provides a sensitivity of 100% (recommended since the goal is not to miss a patient with ROP) and a good specificity (77%).

**TABLE 3:** Estimates from the fitted multivariable logistic regression model; not shown since not significant at the 5% level: CPAP days, intubation, SGA, surfactant, sepsis, IVH, NEC, sex, multiple birth and antenatal steroids.

	Univariable		Multivariable	
	OR (95% CI)	*p*	OR (95% CI)	*p*
Gestational age	0.39 (0.35, 0.43)	< 0.001	0.46 (0.40, 0.52)	< 0.001
Birth weight *z*‐score	0.40 (0.34, 0.48)	< 0.001	0.58 (0.46, 0.73)	< 0.001
Supplemental oxygen (days)	1.03 (1.03, 1.03)	< 0.001	1.01 (1.01, 1.02)	< 0.001
Mechanical ventilation (days)	1.00 (1.00, 1.01)	< 0.001	1.01 (1.00, 1.02)	0.018
Congenital tumour	15.1 (2.28, 59.0)	< 0.001	26.3 (2.71, 189)	0.002
Caesarean section	1.83 (1.17, 3.02)	0.012	1.84 (1.06, 3.36)	0.038

Abbreviations: CI, confidence interval; OR, odds ratio.

All patients requiring ROP treatment would have been detected prior to treatment if screening had been started at 48 days of life but not before 32 + 6 weeks PMA.

**FIGURE 3:**
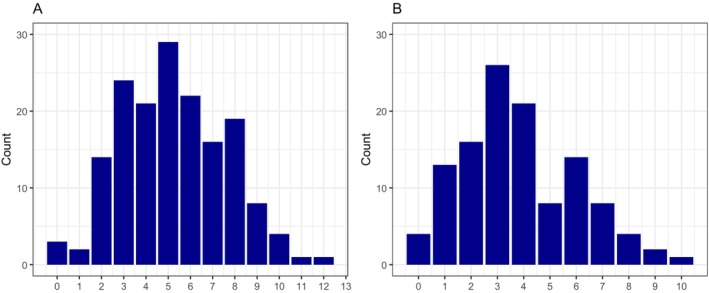
Number of patients requiring ROP treatment in the weeks after initiation of ROP screening. (A) All patients. Start of ROP screening in the seventh week of life (43–49 days) but not before 32 weeks PMA. (B) Patients < 26 0/7 weeks GA. Start of ROP screening in the ninth week of life (57–63 days) but not before 32 weeks PMA.

If weekly screening had been started in the seventh week of life (43–49 days) but not before 32 0/7 weeks PMA, three patients would have required ROP treatment at first screening and two patients in the following week (Figure 3A).

In the subgroup of patients born < 27 0/7 weeks GA, initiation of ROP screening could have been delayed to 60 days of life without missing a patient requiring ROP treatment. If screening had been started in the ninth week of life (57–63 days) but not before 32 0/7 weeks PMA, four patients would have required ROP treatment at first screening and 13 patients in the following week (Figure 3B).

## 4 Discussion

Results of this study show that ROP screening criteria in Switzerland can be optimised. Clinically, the number of screened patients could be reduced by 56% by using four risk factors for patient selection (GA < 27 weeks, birth weight < 1000 g, IVH≥II°, congenital tumour). Logistic regression analyses identified six risk factors which allowed excellent prediction of ROP treatment. It appears feasible to restrict general ROP screening primarily to patients with a birth weight < 1000 g or a GA < 27 weeks unless they have additional risk factors, such as IVH or congenital tumour. Based on the current Swiss data, ROP screening can be started at 48 days of life, but not before 32 + 6 weeks PMA without missing patients who require ROP treatment.

We previously published data on ROP treatment in Switzerland, showing that only 1.2% of patients with a birth weight ≤ 1500 g or a GA < 32 weeks required ROP treatment [18]. The current study shows an incidence of ROP treatment of 1.5%. The small difference can be explained by slightly differing patient populations, specifically the exclusion of patients born ≥ 32 0/7 weeks GA with a birth weight < 1500 g in the current study. Moreover, a higher survival rate of infants born below 25 weeks with the highest risk of severe ROP has been described [19].

The incidence of ROP treatment in Switzerland is significantly lower than in most other high‐income countries, as published in a multi‐national study, comparing ROP incidence across several neonatal networks in high‐income countries [6]. This highlights the need to adapt local screening criteria to avoid under‐ or over‐screening, as both are associated with negative consequences for the infants. While under‐screening increases the risk of undetected ROP and subsequent severe visual impairment or even blindness, screening too many patients leads to increased workload on the ward, increased financial costs and, most importantly, it is associated with unnecessary pain and discomfort for the patients. Since pain perception is associated with impaired neurodevelopment [9, 20, 21], reducing pain is paramount in neonatal care.

Currently, ROP screening criteria vary between hospitals across Switzerland, including differences in GA (30 + 0 to 31 + 6 weeks) or birth weight (1250–1500 g). Additionally, the timepoint of first screening varies between 4 and 6 weeks postnatal age (unpublished data, personal information). To harmonise ROP screening and to base criteria on available evidence, we previously aimed to develop national Swiss screening criteria in 2018 [22]. Available data at that time included only 76 patients requiring ROP therapy, which were too few to develop reliable and safe screening criteria. However, the potential for improved criteria was demonstrated. In fact, to reach a sensitivity of 95% for requiring ROP treatment, only 13% of all patients would have needed ROP screening, while screening all patients was necessary to reach a sensitivity of 100%. In the current study, we additionally included patients born 2016–2022, which allowed to perform similar analyses in a larger patient population and to obtain more reliable results.

To identify the best screening criteria, we assessed 17 risk factors as available from the SNN database. As expected, multiple regression analysis showed that low GA, the duration of supplemental oxygen and mechanical ventilation were independent risk factors for ROP treatment in this Swiss cohort of very preterm infants. These are among the most frequently and most consistently published risk factors for ROP development [23]. The association of ROP with supplemental oxygen has been known since the 1950s [24, 25] and has been confirmed in numerous publications [1, 2, 23, 26].

Our results revealed that a congenital tumour was an independent predictor for ROP treatment. Data on congenital tumours were retrieved from the SSN database and did not include specific screening for tumours, such as ultrasound or other tests. To the best of our knowledge, the association between congenital tumours and ROP treatment has not been published previously. This may be due to the low incidence of congenital tumours, which requires a large sample size to find a significant association. Additionally, many neonatal databases focus on typical morbidities of preterm infants and usually do not include neoplasms. Given the low incidence of congenital tumours and the limited number of patients requiring ROP treatment, our data do not necessitate a recommendation for general screening for congenital tumours. Physiologically, it seems plausible that tumour growth is associated with ROP development, as it may lead to increased levels of growth factors and pro‐angiogenetic metabolites, which may induce vasculopathy of the retina and trigger ROP. Similar associations have been described between ROP and highly vasculated malformations, such as infantile hemangiomas [27, 28, 29]. While the exact mechanisms for the development of hemangiomas are still unclear, it is known to be associated with the β‐adrenergic system and elevated levels of vascular endothelial growth factor (VEGF), which is the main driver for ROP development.

Over the last 15 years, postnatal weight gain has been increasingly studied as a risk factor for ROP development. It has been incorporated in several prediction models, such as the WINROP algorithm [30]. Although initially developed in Sweden, it has been applied to populations of several other countries, with mixed specificity and sensitivity. The SNN collects growth data on weight and *z*‐score at birth and at discharge but does not include data on daily or weekly postnatal growth. This limitation prevented us from incorporating these data in our analyses.

From a clinical perspective, results of our descriptive analyses revealed that all patients requiring ROP treatment would have been detected had the following four criteria been applied: < 1000 g birth weight or < 27 weeks GA or congenital tumour or IVH. Surprisingly, these variables do not include parameters of respiratory support. This contrasts with the majority of prediction models and screening guidelines, which mostly recommend ROP screening for patients requiring any or prolonged or not adequately monitored oxygen therapy [5].

Recent publications have summarised current screening criteria in various countries [10, 31]. Low‐and middle‐income countries mostly recommend screening for patients born at a GA < 32–34 weeks or with a birth weight of < 1500–2000 g. In contrast, guidelines in high‐income countries tend to recommend screening at lower GA and birth weight thresholds, typically around 31 weeks GA and 1250–1500 g birth weight. As mentioned earlier, it appears feasible to restrict general ROP screening in Switzerland to patients with a birth weight < 1000 g or a GA < 27 weeks, or, if patients exceed these cutoffs, in the presence of risk factors for ROP development.

Similar to our approach in Switzerland, the Netherlands developed national screening criteria based on local data. After analysing patients born in 2009, a guideline was published in 2013, which theoretically allowed for a 29% reduction in the number of patients screened [32]. Results of our study show that a much stronger reduction of screening could be achieved in Switzerland, with screening potentially being avoided in 56% of currently screened patients. While in the Netherlands the calculated reduction was successfully implemented in clinical practice, resulting in a 28.4% decrease in ROP screening for a cohort of patients born in 2017 [33], the clinical impact remains to be shown in Switzerland.

A strength of our study is the completeness of the SNN database, which captures 98% of patients born below 32 weeks GA or 1501 g birth weight in Switzerland. Patients at the lower end of GA and birth weight are at the highest risk of requiring ROP treatment and data on this subgroup are expected to be collected at even higher completeness. The large sample size, extended observation period of 17 years in combination with the high data completeness supports the reliability of our findings and the feasibility of developing a national guideline based on these data.

Weaknesses of this study include the relatively low number of patients with treated ROP. Additionally, the long observation period implies that perinatal treatment strategies have evolved over time, which may have influenced the risk of ROP development. Specifically, some Swiss centers adjusted their treatment approach to neonatal care at the limit of viability during the study period, offering more liberal intensive care for extremely preterm infants at lowest GA or birth weight. This resulted in higher survival rates in patients with the highest risk of ROP development [19].

In conclusion, the overall incidence of ROP in Switzerland was similar to previously published data. Detailed analyses of data on Swiss very preterm infants born 2006–2022 demonstrate that a substantial reduction of ROP screening in Switzerland is achievable using more stringent screening criteria. Additionally, the initiation of first screening can be delayed compared to current practice, without compromising patient safety.

## Author Contributions

Dr. Gerull had primary responsibility for protocol development, study design, preparation of the database outcome assessment, preliminary data analysis and writing the manuscript. Dr. Atkinson and Dr. Sanchez were responsible for statistical analyses and interpretation of results. Drs Schuler‐Barazzoni and Barcos Munoz participated in the development of the protocol. Dr. Adams was responsible for data extraction from the SwissNeoNet database. Prof. Gerth‐Kahlert was responsible for ophthalmological aspects of this study. Prof. Schulzke supervised the design and execution of the study. All authors contributed to the interpretation of the results and composition of the final manuscript.

## Consent

The data analysed in this study was collected prospectively in the minimal neonatal dataset (MNDS) registry of the Swiss Neonatal Network (SwissNeoNet). This registry collects routine clinical data of very preterm born infants of all level III neonatal intensive care and all level IIB neonatal intermediate care units of Switzerland. Prior to the enforcement of the Swiss Human Research Act in 2015, data was collected under special authorization by the Federal Health Department and subsequent approval by the Swiss ethics committees: Participating hospitals were allowed to collect data if parents were informed and did not veto data collection. As of 2015, Swiss ethics committees grant approval to each research project individually, if data was collected either via SwissNeoNet informed consent or the individual hospital's general consent. This study's approval carries the number EKNZ 2023–01288.

## Conflicts of Interest

The authors declare no conflicts of interest.

## Data Availability

The data that support the findings of this study are available from the corresponding author upon reasonable request.

## Appendix A Swiss Neonatal Network

We would like to thank the following units for collaborating in the SwissNeoNet: M. Adams (Network Coordinator), University Hospital Zurich; Aarau: Cantonal Hospital Aarau, Children's Clinic, Department of Neonatology (Ph. Meyer, L. Eisenhut); Baden: Cantonal Hospital Baden, Department of Paediatrics (M. Bryant); Basel: University Children's Hospital Basel (UKBB), Department of Neonatology (S.M. Schulzke); Berne: University Hospital Berne, Department of Neonatology (A. Kidszun), Department of Paediatrics (T. Riedel); Biel: Children's Hospital Wildermeth, Department of Paediatrics (M. Gebauer); Chur: Children's Hospital Chur, Department of Neonatology (B. Rogdo); Fribourg: Cantonal Hospital Fribourg, Department of Neonatology (B. Wagner); Geneva: Department of child and adolescent, University Hospital (HUG), Neonatology Unit (R. E. Pfister); Lausanne: University Hospital (CHUV), Department of Neonatology (J.‐F. Tolsa, J. Schneider); Lucerne: Children's Hospital of Lucerne, Neonatal and Paediatric Intensive Care Unit (M. Stocker); Muensterlingen: Cantonal Hospital Muensterlingen, Department of Paediatrics (P. Gessler); Neuchatel: Cantonal Hospital Neuchatel, Department of Paediatrics (B. Laubscher); Sion: Service de pédiatrie de l'Hôpital de Sion (J. Llor); St. Gallen: Cantonal Hospital St. Gallen, Department of Neonatology (A. Malzacher), Children's Hospital St. Gallen, Neonatal and Paediatric Intensive Care Unit (A. Birkenmaier); Winterthur: Cantonal Hospital Winterthur, Department of Neonatology (L. Hegi); Zollikerberg: Hospital Zollikerberg, Neonatal Unit (V. Bernet); Zurich: City Hospital Triemli, Neonatal Unit (M. Tomaske), University Hospital Zurich (USZ), Department of Neonatology (D. Bassler, V. Cannizzaro), University Children's Hospital Zurich, Department of Neonatology (C. Hagmann).
